# Clinical effectiveness of drop-in mental health services in paediatric healthcare settings: a non-randomised multi-site study for children, young people and their families

**DOI:** 10.1186/s12913-025-12681-1

**Published:** 2025-04-14

**Authors:** Anna Roach, Sophie Bennett, Isobel Heyman, Anna Coughtrey, Neha Batura, Lina Gonzalez, Nicki Astle, Rebekah Coates, Jessie Drinkwater, Rebecca Evans, Una Frederick, Michael Groszmann, Steve Jones, Katie McDonnell, Sarah Marley, Amanda Mobley, Abbie Murray, Helena O’Sullivan, Sarah Ormrod, Nyah Patel, Theo Prendegast, Usha Rajalingam, Venkat Reddy, Ameenat Lola Solebo, Isabella Stokes, Emily Webster, Rebecca Webster, Gareth Vinton, Roz Shafran

**Affiliations:** 1 UCL Great Ormond Street Institute of Child Health, 30 Guilford Street, London, WC1N 1EH UK; 2 Great Ormond Street Children’s Hospital, London, WC1N 3JH UK; 3 Department of Psychology, Institute of Psychiatry Psychology & Neuroscience, King’s College London, De Crespigny Park, London, SE5 8AF UK; 4 Cambridge Children’s Hospital Project Team and Paediatric Psychological Medicine Service, Addenbrooke’s Hospital, Hills Road, Cambridge, CB2 0QQ UK; 5 Centre for Global Health Economics, UCL Institute for Global Health, Institute of Child Health, 30 Guilford Street, London, WC1N 1EH UK; 6 Research Department of Primary Care and Population Health, University College London, London, UK; 7 Hinchingbrooke Hospital, Hinchingbrooke Park, Huntingdon, Cambridgeshire PE29 6NT UK; 8 Leeds Teaching Hospital Trust, Paediatric Psychology, E Floor Martin Wing, Leeds General Infirmary, Great George Street, Leeds, LS1 3EX UK; 9 University College Hospitals, 235 Euston Road, London, NW1 2BU UK; 10 Sheffield Children’s Hospital, 3 Northumberland Road, Sheffield, S10 2TT UK; 11 Peterborough City Hospital, Edith Cavell Campus, Bretton Gate, Peterborough, PE3 9GZ UK; 12 UCL Division of Psychology and Language Sciences, 26 Bedford Way, London, WC1H 0AP UK; 13 Child Development Centre, City Care Centre, Cambridgeshire and Peterborough NHS Foundation Trust, Thorpe Road, Peterborough, PE3 6DB UK

**Keywords:** Children, Young people, Mental health, Long term conditions, Implementation

## Abstract

**Background:**

Despite the high prevalence of mental health disorders in children and young people with long-term health conditions, access to timely and effective treatment is often difficult. This study aimed to evaluate the clinical effectiveness of drop-in mental health services for young people with long-term health conditions and their families at six paediatric healthcare settings in England.

**Methods:**

This was a prospective non-randomised single-arm multi-centre interventional study. Young people up to 25 years old with a long-term health condition, and their families were eligible. The primary outcome was the change in the total difficulties score on the Strengths and Difficulties Questionnaire between baseline and 6 months. Interventions provided were standard evidence-based low intensity cognitive-behaviour therapy, onward referral or signposting. Secondary outcomes included quality of life, depression, anxiety, satisfaction with services and cost.

**Results:**

Accessing the drop-in services led to significant reductions in emotional and behavioural symptoms (*p* < 0.01; Cohen’s d = 0.39) and improved quality of life (*p* < 0.01; Cohen’s d = 0.44). Parental depression and anxiety significantly improved (*p* < 0.01; Cohen’s d = 0.30 and d = 0.34).

The average waiting time for an initial assessment was 13.42 days. High levels of satisfaction were reported. The cost per patient was approximately half the estimated cost of a typical course of psychological therapy.

**Conclusions:**

Drop-in mental health services are effective and acceptable and can be delivered at low cost per patient for young people with long term conditions. This model of care is a feasible approach for increasing access to evidence-based mental health treatment in paediatric healthcare settings.

**Trial registration:**

ISRCTN15063954, Registered on 9 December 2022.

**Supplementary Information:**

The online version contains supplementary material available at 10.1186/s12913-025-12681-1.

## Background

Approximately 1.7 million (23%) children and young people (CYP) in England are living with a long-term condition (LTC), defined as any diagnosed health condition lasting for a minimum of three months, for which a cure is unlikely, and which results in limitations in ordinary activities and increased use of health services [1]. CYP with LTCs are significantly more likely to develop mental health symptoms than their healthy peers, with research suggesting around 50% of CYP with LTCs meet diagnostic criteria for at least one mental health disorder, compared with around 16% of the general population [1, 2]. Comorbid physical and mental health conditions have been associated with greater symptom severity and impairment [3], poorer clinical outcomes, and reduced health-related quality of life [4]. In addition, higher rates of parenting stress and anxiety [5] and emotional problems in siblings [6] have been found in families of CYP with a LTC, compared to families without.

Evidence-based treatments for mental health issues, such as cognitive behaviour therapy (CBT), have been shown to be effective in CYP with LTCs [1, 7]. However, practical barriers to accessing mental health treatment persist, often due to the lack of integration between mental and physical health services [8]. One way of providing access to psychological interventions for CYP with LTCs and their families is using a ‘drop-in centre’ located within a paediatric hospital, offering mental health support at point of need. The drop-in centre provided ‘low intensity’ psychological interventions such as guided self-help [9], signposting or referral to appropriate services. This specialist single site model was found to reduce emotional and behavioural symptoms and improve quality of life in CYP with LTCs in a specialist paediatric hospital [10]. It was also found to improve parental and sibling mental health [11, 12], was highly acceptable to families [13] and was cost-effective [14].

Despite many research trials demonstrating effectiveness of mental health interventions, their implementation into clinical practice is rarely considered. Implementation, defined as the methods or techniques used to enhance the adoption and sustainability of a clinical practice, can take up to 17 years [15]. Although the drop-in service model was shown to be effective at a single specialist hospital, it is important to evaluate its effectiveness when rolled out nationally across a range of healthcare settings.

### Objectives

The primary aim of this study was to assess the clinical effectiveness of drop-in mental health services in paediatric healthcare settings. Secondary objectives were to explore family satisfaction and cost per patient of the service.

## Methods

### Trial design

This study was a prospective non-randomised single-arm multi-centre interventional trial. The trial was registered on 9 th December 2022 (ISRCTN15063954) and the protocol published in 2024 [16].

### Participants

Recruitment took place at six paediatric healthcare settings in England: University College London Hospitals, Cambridge and Peterborough Foundation Trust, Hinchingbrooke Hospital, Sheffield Children’s Hospital, Leeds Children’s Hospital and Peterborough City Hospital (see Supplementary Material 1 for more information).

### Eligibility criteria

Individuals had to be a patient, aged between 0 and 25 years, at a participating service for the last 6 months or more, or be a carer/family member/sibling of such patient. They were required to have a mental health need that was interfering with current functioning. Participants who were currently receiving support from paediatric psychology services within their setting were ineligible, however they could be included if on a waiting list.

### Ethics

Written informed consent was obtained for all participants who were aged 16 and above (who had capacity to consent). In the case of children under the age of 16 years, assent was obtained alongside parental consent. Participants could complete consent face-to-face, over the phone or online via Redcap [17].

### Recruitment

The trial was open for recruitment between 16 th November 2022 and 3rd January 2024. Further detail is available in the project protocol [18]. Participants could self-refer or could be referred to the drop-in service through their direct care team.

### Interventions

Once families had consented and completed baseline measures, an initial triage assessment was conducted with a trained practitioner (see Supplementary Material 1). This triage led to the participant receiving one of the three interventions: low intensity CBT, onward referral or signposting. The intervention was delivered to the CYP and/or their parents depending on presenting difficulty, age, participant preference and intellectual ability.

#### Low intensity CBT

Participating services were provided with a list of evidence-based interventions to select from (available in Supplementary Material 2). Treatment was typically 6–8 sessions of guided self-help with less than 6 h of total therapist contact time.

#### Onward referral

These referrals were typically made for cases where there was no evidence-based low intensity CBT intervention e.g., bereavement support or neurodevelopmental assessment. Clinicians were able to refer to internal services in their setting or external services such as local Child and Adolescent Mental Health Services (CAMHS).

#### Signposting

Individuals were signposted to local support groups or charity services where appropriate e.g. domestic violence peer support groups.

### Outcomes

All study measures were completed at baseline after consent and 6 months later. Outcome measures were collected face to face, over the phone or online (depending on participant preference) by a researcher not involved in the delivery of the intervention.

### Primary outcome

#### Strengths and difficulties questionnaire (SDQ)

The primary outcome was the change in total difficulties score on the Strengths and Difficulties Questionnaire (SDQ) reported by parent or child from baseline to 6 months.

The SDQ is a 25-item measure used to measure common emotional and behavioural symptoms in CYP [18–20]. It assesses five subscales: prosocial, hyperactivity, peer problems, emotional problems and conduct problems, in addition to a ‘total difficulties’ score. Each statement is rated on a 3-point Likert Scale of 0 (not true), 1 (somewhat true) and 2 (certainly true). The total difficulties score of the SDQ ranges from 0 to 40 and total scores of 17 and above are above clinical threshold on the parent-reported measures, and 20 and above for self-reported total scores [20]. The SDQ has shown moderate test-retest reliability and good concurrent, convergent and discriminant validity [21].

Self-reported and parent-reported SDQ were completed at baseline and 6 months post-consent. The appropriate form was used depending on the age of the child.

### Secondary outcomes

The SDQ impact subscale and Paediatric Quality of Life Inventory (PedsQL) [19, 22, 23]. Self-reported measures were used where available but the parent-reported measure of the SDQ or PedsQL was substituted as a proxy where necessary. The Patient Health Questionnaire (PHQ- 9) and the Generalized Anxiety Disorder (GAD- 7), for CYP aged 12 years and above which measure depression and anxiety respectively [24, 25]. Parents also completed the PHQ- 9 and GAD- 7 to assess their own mental health. The modified version of the Client Satisfaction Questionnaire (CSQ- 8) [10, 26] and the CHU9D, a paediatric health-related quality of life measure for economic evaluation in healthcare [27].

### Statistical methods

Descriptive statistics for total and subscale scores on each measure at baseline and 6-month follow-up are provided. All descriptive statistics and analyses were undertaken using SPSS statistical analysis software (V.25, IBM). Difference scores were based on the mean change in scores; these changes were tested using paired samples t-tests or Wilcoxon signed rank tests (for non-parametric data) and converted into standardised effect sizes (Cohen’s d). As data were found to be missing not at random, the primary analysis was complete case analysis (CCA) [28].

Self-reported measures were used where available but the parent-reported measure of the SDQ or PedsQL was substituted as a proxy where necessary. This approach ensured maximum use of available data and is reported below as a created “combined” score. Further analysis demonstrating a statistically significant, strong positive correlation between all parent and CYP reported measures, supporting the use of combined scores, is available in Supplementary Material 3. Additional analysis was also conducted looking at parent and CYP reported outcomes separately and is presented in Supplementary Material 4.

For the cost-utility analysis, pre- and post-intervention SDQ scores were converted to health utility scores using an OLS model [29]. The mean group utility values pre- and post-intervention were obtained by mapping the SDQ scores into utility values of CHU9D. Detailed analysis is presented in Supplementary Material 5.

## Results

### Participant flow

**Fig. 1:**
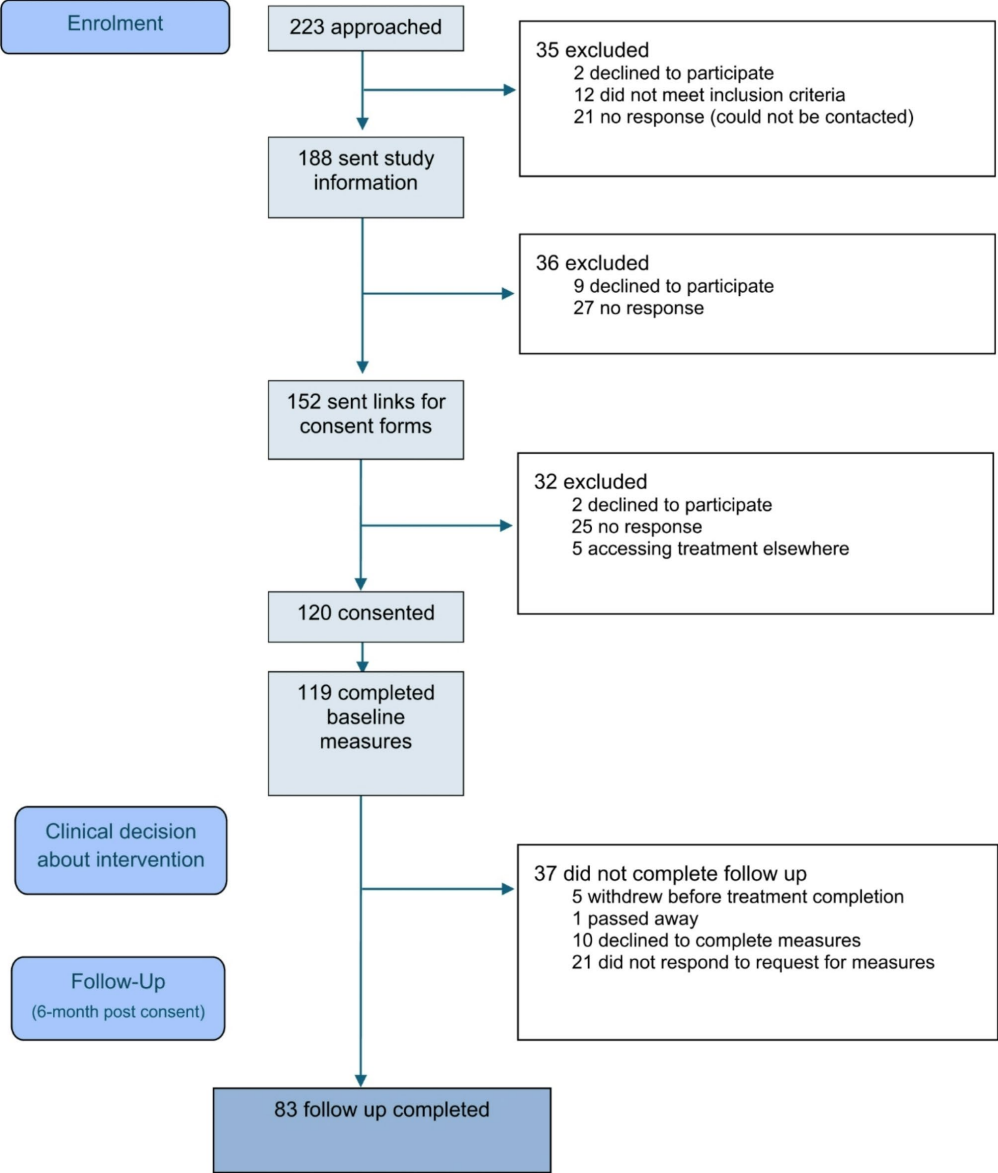
CONSORT diagram

Figure 1 illustrates the flow of participants through the study.

One hundred and twenty families consented to take part in the study across six paediatric healthcare settings. Sixty-six families (55%) were referred by their clinician, and 54 (45%) self-referred to the service. These proportions varied across the different sites.

Participant characteristics are presented in Table 1. The age of CYP in the study ranged from two to 24 years with a mean age of 13.25. Parent-reported measures were completed for CYP age 5 or under, or if requested by the family. The majority of CYP were White British and used English as their first language. Nearly 1/3 of CYP described themselves as having a disability. CYP’s LTC was categorised using the ICD11 coding tool [30].

**Table 1:** Participant and intervention characteristics

Participant characteristics at screening		
*Recruitment per site *(*n*=120)		N
	UCLH	53
	CPFT	9
	Hinchingbrooke	8
	Sheffield	41
	Leeds	4
	Peterborough City Hospital	5
*Children and young people (CYP) *(*n*=120)		
Age in years (*n*=120)		mean(sd), range
		13.25 (4.31), 2-24
		n(%)
Gender (*n*=120)	Female	77 (64.2)
	Male	42 (35.0)
	Non-binary	1 (0.8)
ICD11 condition (*n*=120)	Neoplasms (cancer)	19 (15.8)
	Diseases of the blood or blood-forming organs	3 (2.5)
	Endocrine, nutritional or metabolic diseases	9 (7.5)
	Mental, behavioural or neurodevelopmental disorders	6 (5.0)
	Diseases of the nervous system	20 (16.7)
	Diseases of the visual system	1 (0.8)
	Diseases of the respiratory system	8 (6.7)
	Diseases of the digestive system	5 (4.2)
	Diseases of the skin	2 (1.7)
	Diseases of the musculoskeletal system or connective tissue	3 (2.5)
	Diseases of the genitourinary system	2 (1.7)
	Developmental anomalies	6 (5.0)
	Symptoms, signs or clinical findings, not elsewhere classified	4 (3.3)
	Injury, poisoning or certain other consequences of external causes	1 (0.8)
	Multiple LTCs mentioned	21 (3.3)
	Long covid	6 (5.0)
	Missing LTC	4 (3.3)
Ethnicity (*n*=120)	English, Welsh, Scottish, Northern Irish or British	79 (65.8)
	Any other white background	12 (10.0)
	White and Black Caribbean	2 (1.7)
	White and Black African	1 (0.8)
	White and Asian	2 (1.7)
	Any other mixed or multiple ethnic background	6 (5.0)
	Indian	2 (1.7)
	Pakistani	4 (3.3)
	Any other Asian background	1 (0.8)
	Caribbean	1 (0.8)
	Any other black, black British or Caribbean background	4 (3.3)
	Any other ethnic group	1 (0.8)
	Would rather not disclose	1 (0.8)
First language (*n*=116)	English	110 (94.8)
	Other	6 (5.2)
Disability (*n*=116)	Yes	73 (62.9)
	No	32 (27.6)
	Other	6 (5.2)
	Prefer not to say	5 (4.3)
Employment status (*n*=115)	Employed full time	2 (1.7)
	Employed part time	3 (2.5)
	Self employed	2 (1.7)
	Out of work	2 (1.7)
	Student	91 (75.8)
	Unable to work	4 (3.3)
	Other	10 (8.3)
	Prefer not to say	1 (0.8)
*Parents *(*n*=94)		
Age in years		mean(sd), range
		42.95 (7.29), 26-58
		n(%)
Gender (*n*=94)	Female	87 (92.6)
	Male	7 (7.4)
Ethnicity (*n*=94)	English, Welsh, Scottish, Northern Irish or British	69 (73.4)
	Any other white background	11 (11.7)
	White and Black Caribbean	1 (1.1)
	White and Black African	1 (1.1)
	Any other mixed or multiple ethnic background	3 (3.2)
	Indian	1 (1.1)
	Pakistani	3 (3.2)
	Bangladeshi	1 (1.1)
	Any other Asian background	1 (1.1)
	Caribbean	1 (1.1)
	African	1 (1.1)
	Any other black, black British or Caribbean background	1 (1.1)
First language (*n*=94)	English	82 (87.2)
	Other	12 (12.8)
Disability (*n*=94)	Yes	9 (9.5)
	No	84 (89.4)
	Other	1 (1.1)
Employment status (*n*=94)	Employed full time	30 (31.9)
	Employed part time	25 (26.6)
	Self employed	15 (16.0)
	Out of work	6 (6.4)
	Unable to work	5 (5.3)
	Other	12 (12.8)
	Prefer not to say	1 (1.1)
Marital status (*n*=94)	Married	57 (60.6)
	Single	13 (13.8)
	Living together	12 (12.8)
	Divorced/separated	11 (11.7)
	Not stated/prefer not to say	1 (1.1)
Number of children in house (*n*=94)		mean(sd), range
		2.30 (1.056), 1-5
Assessment and interventions		
Assessment appointment attended^a^		n
	CYP	98
	Parent	8
	Sibling	2
Primary presenting problem (*n*=98)		n (% of CYP who had assessment)
	Anxiety	62 (63.3)
	Low mood	21 (21.4)
	Challenging behaviour	2 (2.0)
	Other	13 (13.3)
Intervention		n (%)
* CYP intervention (n=98)*	Low intensity CBT	57 (58.2)
	Referral to other service	34 (36.1)
	Signposting	4 (4.3)
	Declined intervention^b^	3 (3.2)
* Primary problem (n=57)*	Anxiety	37 (64.9)
	Low mood	10 (17.5)
	Challenging behaviour	2 (3.5)
	Anxiety and depression	7 (12.3)
* Parent intervention (n=8)*	Low intensity CBT	0 (0)
	Referral to other service	3 (37.5)
	Signposting	5 (62.5)

^a^some people declined or did not attend their assessment

^b^some people declined their offer of treatment

Parents’ average age was 42.94 and the majority were female. Most parents were also White British with English as first language. Over half of the parents in the study were employed with the majority working part time.

### Intervention characteristics

The majority of CYP in the study attended an assessment appointment during which information about the individual’s primary presenting problem was collected. 58% of participants were offered 6–8 low intensity CBT sessions, and 65% of this low intensity treatment was for adolescent anxiety. The average wait for an initial assessment was 13.42 days (range 4–34 days).

Only one site offered assessments and intervention for parents (UCLH). Eight parents sought help for their own mental health. Three were referred to existing services and five signposted to external organisations.

#### Missing data

There were consistent significant differences in 6 month follow up completion between families who attended an assessment and the intervention that was received. Since the data were not missing at random, CCA using data only from participants who completed follow up measures, was conducted [28]. Reasons for non-completion are displayed in Fig. 1.

### Main analysis

#### Primary outcome

Emotional and behavioural problems demonstrated a statistically significant decrease from an estimated mean score of 17.68 (6.08) pre-intervention to 15.67 (7.29) at 6 months, post-baseline, a mean decrease of 2.01, 95%CI (0.87, 3.18), *t*(81) = 3.50, *p* < 0.001, *d* = 0.39 (Table 2). Mean total scores at baseline (17.68) were above the clinical threshold whereas 6 moth post-baseline scores (15.67) were below clinical threshold.

**Table 2:** Primary and secondary outcome scores

Outcomes		*N*	Pre	Post	Mean difference (95% CI)	*p*-value	D	df
Primary outcome								
SDQ total difficulties^1^	Mean (sd)	82	17.68	15.57	2.01 (0.87, 3.16)	< 0.001**	0.39	81
Secondary outcomes								
SDQ subscales								
Impact^1^	Mean (sd)	82	3.43	2.89	0.54 (− 0.01, 1.08)^a^	0.05	0.21	81
Emotional^1^	Mean (sd)	82	6.39	5.16	1.23 (0.71, 1.76)	< 0.001**	0.51	81
Conduct^1^	Mean (sd)	82	2.41	2.29	0.12 (− 0.25, 0.49)^a^	0.52	0.07	81
Hyperactivity^1^	Mean (sd)	82	6.28	5.67	0.61 (0.13, 1.09)	0.01*	0.28	81
Peer relationships^2^	Mean (sd)	82	2.60	2.62	0.02 (− 0.40, 0.35)^a^	0.99	0.01	81
Prosocial behaviour^2^	Mean (sd)	82	7.62	7.68	0.06 (− 0.41, 0.29)^a^	0.76	0.04	81
PedsQL								
Total score^1^	Mean (sd)	82	52.44	59.15	− 6.71 (− 10.09, − 3.33)	< 0.001**	0.44	81
Physical health^2^	Mean (sd)	83	60.90	61.16	− 0.26 (− 2.39, 1,86)^a^	0.88	0.03	82
Emotional functioning^2^	Mean (sd)	83	52.47	52.71	− 0.24 (− 2.37, 1.88)^a^	0.40	0.03	82
Social functioning^2^	Mean (sd)	83	69.16	68.07	1.08 (− 0.68, 2.85)^a^	0.22	0.13	82
School functioning^2^	Mean (sd)	83	53.13	55.90	− 2.77 (− 5.75, 0.20)^a^	0.12	0.20	82
Psychosocial health^2^	Mean (sd)	83	58.38	59.02	− 0.64 (− 2.36, 1.07)^a^	0.65	0.08	82
PHQ- 9								
CYP PHQ- 9^2^	Mean (sd)	46	13.28	9.98	3.30 (1.79, 4.82)	< 0.001**	0.65	45
Parental PHQ- 9^2^	Mean (sd)	64	8.17	6.36	1.81 (0.30, 3.33)	0.02*	0.30	63
GAD- 7								
CYP GAD- 7^1^	Mean (sd)	46	10.91	7.70	3.21 (1.73, 4,70)	< 0.001	0.64	45
Parental GAD- 7^1^	Mean (sd)	64	6.89	5.16	1.73 (0.44, 3.03)	0.01*	0.34	63

Means (M), SDs, 95% CIs around the mean difference and effect sizes (d) are shown for all data

*PedsQL* Pediatric Quality of Life Inventory, *PHQ- 9* Patient Health Questionnaire, *GAD- 7* Generalized Anxiety Disorder

*p < 0.05, **p< 0.001

^1^*p* values for paired t-tests are shown for parametric data

^2^*p* values for Wilcooxen signed rank tests are shown for non-parametric data

^a^Confidence intervals pass through 0 and therefore not significant

#### Secondary outcomes

There was also a statistically significant decrease on specific SDQ subscales including emotional symptoms and hyperactivity. All results from the secondary outcomes are displayed in Table 2.

Total quality of life scores on the PedsQL significantly increased from a mean score of 52.44 (18.48) at baseline to 59.15 (21.93), 95%CI (− 10.09, − 3.33), *t*(81) = − 3.95, *p* < 0.001, *d* = 0.44 (Table 2). In addition, when looking at parent and CYP reported outcomes separately (in Supplementary Material 3) all subscales on the PedsQL showed significant increases from baseline to 6 month follow up.

The non-parametric Wilcoxon signed rank test was used to determine whether there was a significant change in depression scores. For CYP over the age of 12 (*n* = 46), PHQ- 9 depression scores decreased from 13.28 (6.12) to 9.98 (6.48), *Z*=− 3.96, *p* < 0.001, *d* = 0.65 from baseline to 6 month follow up. Scores moved from the “moderate” clinical range to “mild”.

There was also a significant decrease in parental depression (*n* = 64) from 8.17 (6.35) to 6.36 (6.00), *Z*= − 2.42, *p* = 0.02, *d* = 0.30 from baseline to 6 month follow up. Scores remained within the “mild” range.

There was a significant decrease in anxiety symptoms for CYP with child-reported anxiety scores (*n* = 46) decreasing from 10.91 (5.40) to 7.70 (5.98), 95% CI (1.73, 4.70), *t*(45) = 4.37, *p* < 0.001, *d* = 0.64. As scores above 10 are considered to be within the clinical range, average follow-up anxiety scores for CYP have been found to no longer meet clinical threshold.

Parental self-reported anxiety scores (*n* = 64) decreased from 6.89 (5.71) to 5.16 (4.72), 95% CI (0.44, 3.03), *t*(63) = 2.68, *p* = 0.01, *d* = 0.34.

As the results indicated a significant difference between baseline and 6-month post consent outcomes for those who accessed the drop-in services, post-hoc t-tests for parametric data were conducted to investigate if this change was being driven by the specific intervention that CYP received.

Participants receiving low intensity CBT showed a significant difference in SDQ scores from a mean score of 15.64 (5.60) at baseline to 13.75 (6.56) at 6 month post-consent, a mean difference of 1.89, 95% CI (0.33, 3.46), *t*(47) = 2.26, *p* = 0.02, *d* = 0.35 (see Table 3). For those referred to existing services, there was also a significant improvement when comparing mean scores.

**Table 3:** Differences in SDQ scores across different interventions

Outcomes		*N*	Pre	Post	Mean difference (95% CI)	*p*-value	D	df
LI CBT only								
SDQ total difficulties	Mean (sd)	48	15.64 (5.60)	13.75 (6.56)	1.89 (0.33, 3.46)	0.02*	0.35	47
Referral only								
SDQ total difficulties	Mean (sd)	22	20.31 (5.43)	17.95 (7.70)	2.36 (0.15, 4.58)	0.04*	0.47	21
Signposting only								
SDQ total difficulties	Mean (sd)	3	24.33 (2.52)	22.33 (7.77)	2.00 (− 22.33, 18.33)	0.75	0.24	3

##### CSQ- 8

Seventy-seven families completed the modified CSQ- 8. 30 reports were from the same family, with 18 CYP-report only and 29 parent-report only. Responses and proportions split by CYP and parent-report are shown in Supplementary Material 4. Both CYP and parents indicated they would “Totally” (median = 5) recommend the project to a friend. The only question with a lower median score was “whether the information and support received made any difference to you/your child’s physical health”, which CYP reported, 2, “only a little” and parent reported, 3, “somewhat”.

##### Cost per patient

Intervention costs were calculated overall and per site and baseline and 6 month follow-up SDQ scores were converted to CHU9D health utilities. The total cost of the drop-in service per patient was £358.56, and analysis revealed an average cost per unit increase on the CHU9D of £9,743.60, and an average cost per unit decrease on the SDQ of £183.88. Full analysis including cost per site are presented in Supplementary Material 5.

### Harms

None were reported.

## Discussion

Overall, there were statistically significant improvements in both mental health symptoms and quality of life for CYP who accessed the drop-in mental health services across the different paediatric healthcare settings. The effect sizes ranged from small to moderate. This improvement extended to parental mental health as their own depression and anxiety symptoms also significantly decreased across the 6-month period. Patients were highly satisfied with the service, and the cost per patient was approximately half the estimated cost of a typical course of psychological therapy [31].

The findings were comparable to those of the earlier study at a single specialist paediatric centre [10], indicating that it is possible to implement this service model across paediatric healthcare settings and maintain effectiveness. The services acted as a single point of access for CYP and their families to receive help at the time of need, with average wait time for an assessment appointment being less than two weeks.

Total scores on the SDQ and the subscale of emotional symptoms subscale showed a statistically significant decrease. There were improvements (i.e. decrease in scores) for the other subscales of the SDQ however these changes in score were not statistically significant. This may be because the majority of the sample were adolescents who completed anxiety treatment, and thus behavioural symptoms were not targeted and did not change. There was also a significant improvement in quality of life when using the total PedsQL score.

An important question to ask is whether the statistical significance of the findings translates into clinical significance. Previous research indicates that the odds of psychiatric disorder decrease by 40% for each 2-point decrease in the parent-reported SDQ [21]. This study saw a 2.01-point decrease in SDQ score from baseline to 6 month follow up thus suggesting the drop-in service had a meaningful and clinically significant effect.

Another question is whether the mental health of the participants improved as a result of an improvement in physical health, rather than the drop-in service. Although scores on the PedsQL physical subscale showed some improvement, responses on the CSQ- 8 indicated that physical health had not changed significantly over the course of the study. Many of the CYP in the trial have ‘chronic’ LTCs with limited or very slow recovery, thus symptoms may not change in a 6 month period.

Results also indicated statistically significant decreases in anxiety and depression scores for parents, again replicating the original findings [11]. This may be interpreted alongside existing research which demonstrates that child mental health and parental mental health are associated [32]. Parents of CYP with LTCs are known to have elevated mental health needs compared to the general population and changes in parental mental health may be attributable to improvements in their CYP’s mental health with some studies suggesting it may not be necessary to treat CYP and parents separately to affect significant positive change for both [33].

The central findings from this study are first that the drop-in model can be implemented and effective in multiple paediatric healthcare settings, and second that standard evidence-based low intensity interventions benefit CYP with mental health difficulties in the context of chronic illness.

This trial had important strengths. This study met the recruitment target and included a large number of families, across the different settings with CYP living with range of LTCs. The study used a range of validated outcome measures, including both self-reported and parent-reported measures which are commonly used in routine practice which could facilitate comparisons between the study sample and those seen by local services in future analysis.

The primary limitation of the study was the lack of control group. Although the results suggest that drop-in mental health services are clinically effective, this change cannot be specifically attributed to attending the drop-in services, as participants may have improved with time regardless. Future research should conduct a randomised control trial where the intervention (a drop-in service providing an assessment and delivering a range of interventions including low intensity CBT) is compared with CYP on a wait list (to control for passage of time), or an active control. An additional reflection was the attrition rate, as 70% of the sample completed follow up measures. The main reasons for this were that participants did not respond to requests, and this appeared to be particularly true if they had not received an intervention. Incentives to encourage participants to complete questionnaires may have been helpful in improving the response rate. Furthermore, a longer follow up time period would be beneficial to establish if the improvements in emotional and behavioural symptoms, and/or quality of life, are maintained and to account for cognitive change which may take longer than 6 months.

## Conclusion

Overall, this study indicates that drop-in mental health services are effective and acceptable and can be delivered at low cost per patient. The findings point to the feasibility and effectiveness of rolling out drop-in mental health services which act as single point of access in paediatric healthcare settings to improve access to essential services for CYP with LTCs.

## Supplementary Information

Supplementary Material 1.

Supplementary Material 2.

Supplementary Material 3.

Supplementary Material 4.

Supplementary Material 5.

## Data Availability

De-identified datasets used and/or analysed during the current study are available from the corresponding author on reasonable request.

## Abbreviations

CYP: Children and young people
LTC: Long term condition
CBT: Cognitive behaviour therapy
SDQ: Strengths and Difficulties Questionnaire
PHQ- 9: Patient Health Questionnaire
PedsQL: The Pediatric Quality of Life Inventory
GAD- 7: Generalized Anxiety Disorder Assessment
CSQ- 8: Client Satisfaction Questionnaire
CHU9D: Child health utility instrument
