# Supplementary material for: Clinical effectiveness of drop-in mental health services in paediatric healthcare settings: a non-randomised multi-site study for children, young people and their families
Source: BMC Health Serv Res. 2025 Apr 14;25:546. doi: 10.1186/s12913-025-12681-1 (PMC11998343; doi:10.1186/s12913-025-12681-1)
Supplement: Supplementary file 1 — Supplementary Material 1. [file 12913_2025_12681_MOESM1_ESM.docx]

## Supplementary Material 1: Project pathway for each site

| **SITE:** | **UCLH** | **CPFT** | **Hinchingbrooke** | **Sheffield** | **Leeds** | **PCH** |
| --- | --- | --- | --- | --- | --- | --- |
| Site practitioner(s) | Senior low intensity practitioner (EMHP) and trainee CWPs | Clinical psychologist | Clinical nurse specialist and consultant paediatrician | Senior CWPs | Assistant psychologist | Assistant psychologist and clinical nurse specialist |
| Supervision | Clinical psychologist in paediatric psychology team | Clinical psychologist in paediatric psychology team | Clinical psychologist from research team | Clinical psychologist in paediatric psychology team | Clinical psychologist in paediatric psychology team | Clinical psychologist from research team |
| Conditions of CYP | All conditions | All conditions | Epilepsy | All physical health conditions | Long covid | Epilepsy |
| Project pathway | Self-referral via project posters and clinician referrals direct to study team. | Self-referral via project posters and clinician referrals direct to study team. | Clinician referrals from site study practitioners direct to study team | Self-referrals only from posters and/or leaflets in the hospital. | Formed part of the triage clinic meeting. Families on a waitlist for psychology support were offered low intensity interventions | Clinician referrals from site study practitioners direct to study team |
