# Supplementary material for: Clinical effectiveness of drop-in mental health services in paediatric healthcare settings: a non-randomised multi-site study for children, young people and their families
Source: BMC Health Serv Res. 2025 Apr 14;25:546. doi: 10.1186/s12913-025-12681-1 (PMC11998343; doi:10.1186/s12913-025-12681-1)
Supplement: Supplementary file 2 — Supplementary Material 2. [file 12913_2025_12681_MOESM2_ESM.docx]

## Supplementary Material 2: Low intensity CBT interventions

| **Assessment** |
| --- |
| 1. Assessment Guide For Clinicians (Bennett et al., 2022) |
| **Anxiety** |
| 1. Helping Your Child with Fears and Worries: A Self-Help Guide for Parents (Creswell and Willets, 2019) 2. Getting to Grips with Anxiety: A Guided Self-Help Workbook (Barker et al., 2021) |
| **Depression** |
| 1. Behavioural Activation for Young People with Low Mood: Guided Self-Help Manual (Maiden, N.D.) 2. Brief Behavioural Activation for Adolescent Depression: A Clinician's Manual and Session-by-Session Guide (Reynolds and Pass, 2020). |
| **Challenging behaviour** |
| 1. Guided Self Help for Common Behaviour Problems (Woolgar et al., 2022) 2. The Incredible Years: Trouble Shooting Guide for Parents of Children Aged 3-8 Years (Webster-Stratton, 2005) |

Barker, H., Payne, S., Williams, S. and Bowyer, L. (2021). Getting to grips with Anxiety A Guided Self-Help Workbook. https://www.kings-cyp.com/media/getting-to-grips-with-anxiety_update-march-2021.pdf

Bennett, S., Myles-Hooton, P., Schleider, J., & Shafran, R. (2022). Oxford Guide to Brief and Low-intensity Interventions for Children and Young People. Oxford University Press.

Creswell, C., & Willetts, L. (2019). Helping Your Child with Fears and Worries 2nd Edition: A Self-Help Guide for Parents. Hachette UK.

Maiden, Z. (n.d.). Behavioural Activation (BA) for Young People with Low Mood: Guided Self-Help Manual. https://www.kings-cyp.com/media/ba-gsh-manual-final.pdf

Reynolds, S., & Pass, L. (2020). Brief Behavioural Activation for Adolescent Depression: A Clinician’s Manual and Session-by session Guide. Jessica Kingsley Publishers.

Webster-Stratton, C., Mostyn, D., & Marie, J. S. (2005). The Incredible Years: A trouble-shooting guide for parents of children aged 2-8 years. Seattle, WA: Incredible years.

Woolgar, M., Bengo, C., & Dawson, S. (2022). Guided Self Help for Common Behaviour Problems. https://www.kings-cyp.com/media/behaviours-that-challenge-manual-2022.pdf
