# Supplementary material for: Clinical effectiveness of drop-in mental health services in paediatric healthcare settings: a non-randomised multi-site study for children, young people and their families
Source: BMC Health Serv Res. 2025 Apr 14;25:546. doi: 10.1186/s12913-025-12681-1 (PMC11998343; doi:10.1186/s12913-025-12681-1)
Supplement: Supplementary file 3 — Supplementary Material 3. [file 12913_2025_12681_MOESM3_ESM.docx]

## Supplementary Material 3: Use of combined scores:

Bivariate correlations using Pearson’s r and Spearman's rho coefficients (for non-parametric data) were used to explore the association between parent and child reported outcomes (De Winter et al., 2016). As shown in Table 6-13 there was a statistically significant, strong positive correlation between all parent and child reported measures. This supports the use of combined scores.

Table 6‑13- Parent and child correlations

|  |  | **r/rho** | **p** |
| --- | --- | --- | --- |
| SDQ at baseline | | .725^a^ | <.001 |
| SDQ at 6 month follow up | | .853^b^ | <.001 |
| PedsQL at baseline | | .733^a^ | <.001 |
| PedsQL at 6 month follow up | | .793^b^ | <.001 |

^a^ Pearsons r, ^b^ Spearmans rho
