# Supplementary material for: Clinical effectiveness of drop-in mental health services in paediatric healthcare settings: a non-randomised multi-site study for children, young people and their families
Source: BMC Health Serv Res. 2025 Apr 14;25:546. doi: 10.1186/s12913-025-12681-1 (PMC11998343; doi:10.1186/s12913-025-12681-1)
Supplement: Supplementary file 4 — Supplementary Material 4. [file 12913_2025_12681_MOESM4_ESM.docx]

## Supplementary Material 4: Analysis conducted using parent and CYP reported outcomes separately

Table 3 - Primary outcome scores for Parent and CYP separately

| Outcomes |  | N | Pre | Post |  | Mean difference  (95% CI) | p-value | D | df |  |
| --- | --- | --- | --- | --- | --- | --- | --- | --- | --- | --- |
| **PRIMARY OUTCOME** | | | | | | | | | | |
| Parent-reported SDQ total difficulties | Mean (sd) | 53 | 17.53 | 15.55 |  | 1.98 (0.60, 3.36) | .006** | 0.40 | 52 |  |
| Self-reported SDQ total difficulties | Mean (sd) | 63 | 15.75 | 13.86 |  | 1.89 (0.57, 3.21) | .006** | 0.36 | 62 |  |

SDQ, Strengths and Difficulties Questionnaire
Means (M), SDs, 95% CIs around the mean difference, p values for paired t-tests and effect sizes (d) are shown for all participants included in the analysis.
*p<0.05, **p<0.01, ***p<0.001

Table 4- Secondary outcomes for Parent and CYP separately

| Outcomes |  | N | Pre | Post |  | Mean difference  (95% CI) | p-value | D | df |  |
| --- | --- | --- | --- | --- | --- | --- | --- | --- | --- | --- |
| **SECONDARY OUTCOMES** | | | | | | | | | | |
| Parent reported SDQ subscales |  |  |  |  |  |  |  |  |  |  |
| Impact^2^ | Mean (sd) | 63 | 15.75 | 13.86 |  | 1.89 (0.57, 3.21) | .01** | 0.36 | 62 |  |
| Emotional^1^ | Mean (sd) | 63 | 6.02 | 4.44 |  | 1.58 (0.90, 2.24) | <.001*** | 0.59 | 62 |  |
| Conduct^2^ | Mean (sd) | 63 | 2.06 | 2.03 |  | 0.03 (-0.28, 0.34)^a^ | .93 | 0.03 | 62 |  |
| Hyperactivity^1^ | Mean (sd) | 63 | 5.10 | 4.89 |  | 0.21 (-0.31, 0.73)^a^ | .43 | 0.10 | 62 |  |
| Peer relationships^1^ | Mean (sd) | 63 | 2.57 | 2.49 |  | 0.08 (-0.36, 0.51)^a^ | .72 | 0.05 | 62 |  |
| Prosocial behaviour^2^ | Mean (sd) | 63 | 7.57 | 8.62 |  | 1.05 (-2.99, 0.89)^a^ | .29 | 0.14 | 62 |  |
| Self- reported SDQ subscales | | | | | | | | | | |
| Impact^1^ | Mean (sd) | 53 | 2.92 | 2.32 |  | 0.60 (-0.06, 1.27)^a^ | 0.07^a^ | 0.25 | 52 |  |
| Emotional^1^ | Mean (sd) | 53 | 6.66 | 5.66 |  | 1.00 (0.32, 1.68) | .005** | 0.40 | 52 |  |
| Conduct^1^ | Mean (sd) | 53 | 2.23 | 1.94 |  | 0.29 (-0.19, 0.77)^a^ | .24 | 0.16 | 52 |  |
| Hyperactivity^1^ | Mean (sd) | 53 | 6.08 | 5.42 |  | 0.66 (0.11, 1.21) | .02* | 0.33 | 52 |  |
| Peer relationships^2^ | Mean (sd) | 53 | 2.57 | 2.64 |  | 0.07 (-0.51, 0.36)^a^ | .58 | 0.05 | 52 |  |
| Prosocial behaviour^2^ | Mean (sd) | 53 | 7.77 | 8.00 |  | 0.23 (-0.71, 0.25)^a^ | .33 | 0.13 | 52 |  |
| **PedsQL** | | | | | | | | | | |
| Parent reported PedsQL | | | | | | | | | | |
| Total score^2^ | Mean (sd) | 64 | 52.51 | 61.72 |  | -9.21 (-13.31, -5.11) | <.001*** | 0.56 | 63 |  |
| Physical health^1^ | Mean (sd) | 64 | 56.59 | 64.21 |  | -7.62 (12.81, -2.42) | .005** | 0.36 | 63 |  |
| Emotional functioning^2^ | Mean (sd) | 64 | 40.63 | 53.98 |  | -13.36 (-18.55, -8.17) | <.001*** | 0.64 | 63 |  |
| Social functioning^1^ | Mean (sd) | 64 | 65.00 | 70.16 |  | -5.16 (-10.54, 0.23)^a^ | .06* | 0.23 | 63 |  |
| School functioning^2^ | Mean (sd) | 64 | 47.81 | 58.52 |  | -10.70 (-16.36, -5.04) | <.001*** | 0.47 | 63 |  |
| Psychosocial health^2^ | Mean (sd) | 64 | 50.99 | 60.88 |  | -9.90 (-14.17, -5.62) | <.001*** | 0.58 | 63 |  |
| Self-reported PedsQL | | | | | | | | | | |
| Total score^1^ | Mean (sd) | 58 | 51.78 | 61.00 |  | -9.22 (-13.20, -5.23) | <.001*** | 0.61 | 57 |  |
| Physical health^1^ | Mean (sd) | 58 | 57.06 | 61.29 |  | -4.23 (-7.91, -0.54) | .01* | 0.56 | 57 |  |
| Emotional functioning^1^ | Mean (sd) | 58 | 41.12 | 55.09 |  | -13.97 (-19.37, -8.57) | </001*** | 0.68 | 57 |  |
| Social functioning^1^ | Mean (sd) | 58 | 63.10 | 69.48 |  | -6.38 (-11.60, -1.16) | .01* | 0.32 | 57 |  |
| School functioning^1^ | Mean (sd) | 58 | 46.12 | 57.59 |  | -11.47 (-17.86, -5.07) | <.001*** | 0.47 | 57 |  |
| Psychosocial health^1^ | Mean (sd) | 58 | 50.11 | 60.90 |  | -10.78 (-15.45, -6.11) | <.001*** | 0.61 | 57 |  |

PedsQL, Pediatric Quality of Life Inventory; PHQ-9, Patient Health Questionnaire; GAD-7, Generalized Anxiety Disorder
Means (M), SDs, 95% CIs around the mean difference and effect sizes (d) are shown for all data

*p<0.05, **p<0.01, ***p<0.001

^1^ p values for paired t-tests are shown for parametric data

^2^ p values for Wilcooxen signed rank tests are shown for non-parametric data

^a^Confidence intervals pass through 0 and therefore not significant
