# Supplementary material for: Clinical effectiveness of drop-in mental health services in paediatric healthcare settings: a non-randomised multi-site study for children, young people and their families
Source: BMC Health Serv Res. 2025 Apr 14;25:546. doi: 10.1186/s12913-025-12681-1 (PMC11998343; doi:10.1186/s12913-025-12681-1)
Supplement: Supplementary file 5 — Supplementary Material 5. [file 12913_2025_12681_MOESM5_ESM.docx]

## Supplementary Material 5: Health economic analysis:

**Mapping SDQ scores for cost-utility analysis**

The mean group utilities were obtained using the first Ordinary Least Squares (OLS) algorithm, which employs five subscales from the SDQ to calculate the utility values.

The formula used is as follows: **Utility = 0.880 + (-0.019 × emotion) + (-0.009 × conduct) + (-0.001 × hyperactivity) + (-0.008 × peer problems) + (0.005 × prosocial).**

This method ensures a comprehensive evaluation by considering multiple dimensions of the SDQ, thereby providing accurate and robust utility values for subsequent economic analyses.

**Converting SDQ scores:**

**Child SDQ:**

| **Utility Conversion** | | **Difference** | **Discounted Post-Treatment** |
| --- | --- | --- | --- |
| Pre-treatment | Post-treatment |  |  |
| **0.746** | **0.770** | **0.0236** | **0.0236** |

**Parent SDQ:**

| **Utility Conversion** | | **Difference** | **Discounted Post-Treatment** |
| --- | --- | --- | --- |
| Pre-treatment | Post-treatment |  |  |
| **0.758** | **0.796** | **0.0378** | **0.0378** |

**Combined SDQ:**

| **Utility Conversion** | | **Difference** | **Discounted Post-Treatment** |
| --- | --- | --- | --- |
| Pre-treatment | Post-treatment |  |  |
| **0.742** | **0.779** | **0.0368** | **0.0368** |

**Discounting**

In this intervention, the outcomes were discounted at 0 years because the follow-up period was only six months. However, for follow-up periods extending beyond 12 months, discounting should be applied to account for the time preference of costs and health outcomes.

This discounting process is necessary because future costs and health outcomes are typically valued less than those in the present. By expressing the results as streams of health outcomes and costs over time and applying a discount factor to each value, we can aggregate these to provide a 'present value' for each stream.

**Cost per site:**

Intervention costs were calculated by asking the different clinicians involved over the duration of the intervention. Unit costs for staff were taken from the most recent version of the Personal Social Services Resource Unit (PSSRU)’s unit costs of health and social care, as there were different grades of therapists reported. Training and supervision costs were included as well.

Additionally, the number of hours of training per person was recorded. If more people attended specific trainings, average training costs per site was lower. For example, where the in-person training can accommodate up to 20 people, this means 20 people can be trained without additional costs. When training is delivered online, there is only a fixed cost of delivering training, as there is no limit on how many people can attend. As explored Chapter 5, the training, although encouraged, was not mandatory. This resulted in pronounced training cost differences amongst sites. Supervision was mandatory for all therapists and therefore supervision costs were included for every site.

A breakdown of all costs is shown in Table A1 and cost per site is presented in Table A2.

Table D1: Cost of Lucy Project

| **Total cost of project** | **£43,027.75** | **Average cost per unit increase in CHU9D** | **Average cost per unit increase in SDQ** |
| --- | --- | --- | --- |
| **Total cost of lucy per patient** | £358.56 | £9,743.60 | £183.88 |
| **Cost of supervision** | £10,169.70 |  |  |
| **Total cost of training** | £ 4,213.05 |  |  |
| *Cost of trainers* | *£205.05* |  |  |
| *Cost of attending training* | *£4,008.00* |  |  |
| **Cost of delivering Lucy project** | £28,645.00 |  |  |

Table D2: Cost Per Site

| **Site** | **Cost per site** | **Number of patients receiving low intensity CBT** | **Cost per site and per patients seen** |
| --- | --- | --- | --- |
| UCLH | £16,254.50 | 44 | £369.42 |
| Sheffield | £9,660.00 | 32 | £301.88 |
| CPFT | £3,815.42 | 9 | £423.94 |
| Hinchingbrooke | £7,250.26 | 5 | £1,450.05 |
| Leeds | £2,397.26 | 3 | £799.09 |
| Peterborough | £3,650.30 | 3 | £1,216.77 |

The analysis revealed an average cost per unit increase on the CHU9D of £9,743.60, and an average cost per unit decrease on the SDQ of £183.88. As mentioned previously, there are clinical implications of a two-point decrease in SDQ score (odds of psychiatric disorder decreasing by 40%) and this analysis suggests possible economic implication for one point change too.

*Limitations*

The SDQ primarily measures symptom severity. Therefore, choosing an alternative measure that prioritizes quality of life would have made the analysis more accurate.

Data was collected only for a 6-month period with a small sample size of 120 participants, of whom 40 were lost to follow-up, leaving data for only 80 participants. To ensure robust conclusions, extensive sensitivity analysis and data on the remaining participants are necessary.

Furthermore, the outcomes in this analysis have not been converted into Quality-Adjusted Life Years (QALYs). This limitation should be addressed in future research to better inform resource allocation and priority setting in healthcare. Converting outcomes into QALYs could provide a more comprehensive understanding of the intervention's impact on quality and quantity of life, thus enhancing the economic evaluation and aiding in more effective decision-making.

*Conclusion*

In conclusion, while the Lucy project shows promise in improving health utilities and SDQ scores in paediatric patients with LTCs, the high cost per unit increase in CHU9D suggests the need for a careful consideration of resource allocation. The intervention could potentially be cost-effective, but further analysis is needed to confirm this. Future research should focus on longer-term outcomes and include a broader set of measures prioritizing quality of life to provide a more comprehensive evaluation.
